# Enhancing memory retrieval in generative agents through LLM-trained cross attention networks

**DOI:** 10.3389/fpsyg.2025.1591618

**Published:** 2025-05-07

**Authors:** Chuanyang Hong, Qingyun He

**Affiliations:** ^1^School of Computing and Artificial Intelligence, Southwestern University of Finance and Economics, Chengdu, China; ^2^School of Finance and Economics, Anhui Science and Technology University, Bengbu, China

**Keywords:** artificial intelligence (AI), large language models (LLMs), generative agents, memory retrieval, attention mechanism

## Abstract

**Introduction:**

The surge in the capabilities of large language models (LLMs) has propelled the development of Artificial General Intelligence (AGI), highlighting generative agents as pivotal components for emulating complex AI behaviors. Given the high costs associated with individually training LLMs for each AI agent, there is a critical need for advanced memory retrieval mechanisms to maintain the unique characteristics and memories of individual AI agents.

**Methods:**

In this research, we developed a text-based simulation of a generative agent world, constructing a community with multiple agents and locations in which certain levels of interaction were enabled. Within this framework, we introduced a novel memory retrieval system using an Auxiliary Cross Attention Network (ACAN). This system calculates and ranks attention weights between an agent's current state and stored memories, selecting the most relevant memories for any given situation. In a novel approach, we incorporated LLM assistance, comparing memories retrieved by our model with those extracted using a base method during training, and constructing a novel loss function based on these comparisons to optimize the training process effectively. To our knowledge, this is the first study to utilize LLMs to train a dedicated agent memory retrieval network.

**Results:**

Our empirical evaluations demonstrate that this approach substantially enhances the quality of memory retrieval, thereby increasing the adaptability and behavioral consistency of agents in fluctuating environments.

**Discussion:**

Our findings not only introduce new perspectives and methodologies for memory retrieval in generative agents but also extend the utility of LLMs in memory management across varied AI agent applications.

## 1 Introduction

The release of GPT-4 by OpenAI has demonstrated the impressive capabilities of large language models (LLMs) and their potential for Artificial General Intelligence (AGI). Consequently, various Artificial Intelligence (AI) applications based on LLMs have made significant advancements across different fields. Among these, personalized AI agents that simulate human behavior have garnered increasing attention and are considered a crucial pathway toward AGI (Xi et al., 2023).

The concept of an agent refers to entities possessing desires, beliefs, intentions, and the ability to take actions (Zalta et al., 1995). Currently, the goal of LLM based generative agents is to simulate believable human behavior, creating more personalized AI. This requires AI not only to simulate human behavior at a single point in time but to ensure long-term coherence. Such AI would be better suited by architectures that manage ever-growing memories as new interactions, conflicts, and events arise and fade over time while handling cascading social dynamics that unfold between multiple agents (Park et al., 2023).

Therefore, personalized AI requires not only the general intelligence provided by LLMs but also long-term personalized memories that are private, extensible, and explainable to the user. Additionally, it requires an efficient method to retrieve these relevant memories based on the current context faced by the agent.

To achieve this goal, the ideal approach would be to train a dedicated LLM for each agent. However, considering the complexity of LLM training (Yang et al., 2024) and the practical demands of a large variety and number of agents, this approach is impractical. Therefore, the common practice is to store the agent's memories externally and provide the necessary memories to the LLM in the form of linguistic feedback during decision-making (Shinn et al., 2023).

In this approach to implementing agents, the method of memory retrieval becomes critically important. The ability to extract memories relevant to the current context faced by the agent will directly determine how well the agent's behavior can simulate real human actions. Common memory retrieval methods include temporal decay ranking, evaluation of memory importance, vector similarity matching, and combinations of these techniques (Park et al., 2023). However, these existing methods still have significant limitations in matching the complex correlations between the agent's current context and the memories stored in the memory bank.

**Figure 1:**
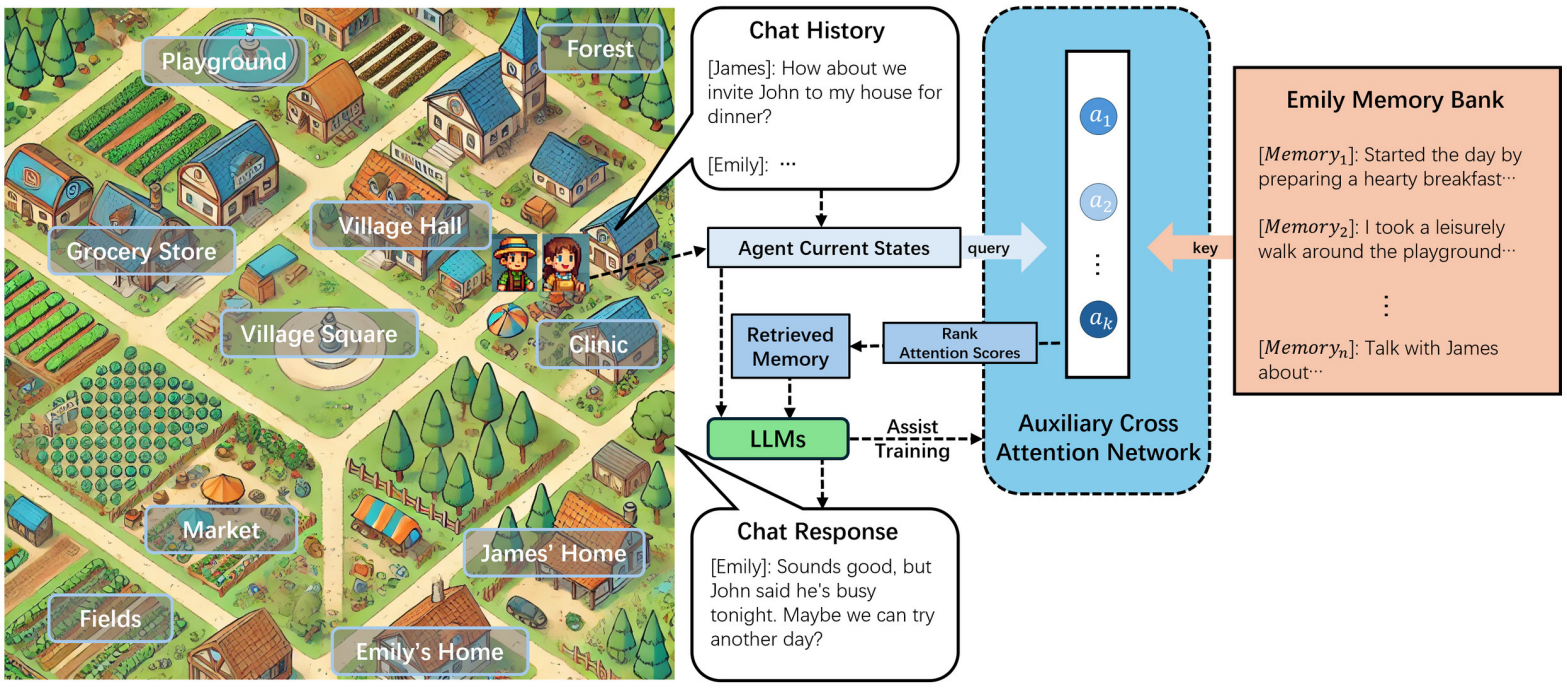
Illustration of the simulated world and memory retrieval process.

Faced with this challenge, we developed a text-based generative agent simulation environment featuring multiple characters and locations, as depicted in Figure 1. This simulation framework enabled the modeling of agents with diverse characteristics, including varying ages, genders, identities, professions, and personalities, all portrayed by LLMs. These agents operated within a virtual village, residing in their respective homes and interacting in public spaces. Through extended simulations and systematic observation of the agents' behaviors and feedback, we sought to evaluate the impact of different memory retrieval methods on the agents' ability to simulate human behavior effectively.

Building on this foundation, we propose an innovative memory retrieval method designed for generative agents that simulate human-like interactions. This method uses an Auxiliary Cross Attention Network (ACAN) to optimize memory retrieval. Inspired by the self-attention mechanism described in Vaswani et al. (2017), ACAN transforms the agent's current state and observed context into a query vector. This query is compared with stored memories in the memory bank, which are represented as key-value pairs. The attention mechanism calculates scores by aligning the query with the memory keys, and the attention weights are ranked. Based on these ranked attention scores, the most relevant memories are selected as the retrieved memory set.

The retrieved memories are combined with the agent's current state and input into the LLM to guide the agent's behavior. To train this network and enhance its ability to simulate the human memory retrieval process, we innovatively introduced the use of LLMs to assist in network training. By comparing the memories retrieved by this network with traditional memory retrieval methods, allowing the LLM to evaluate and score the quality of the retrieved memories based on the agent's current state. These scores are then incorporated into the custom loss function to guide the training of the ACAN. This method ensures that the network is updated in a way that better reflects human-like memory retrieval patterns. To the best of our knowledge, this is the first approach that integrates LLMs into the training process of a memory retrieval network for agents.

Compared to existing static memory retrieval algorithms, the ACAN approach introduces dynamic improvements by incorporating the agent's historical memory formation process with a cross-attention mechanism that is optimized through LLM feedback. Our experiments demonstrate that ACAN substantially outperforms traditional methods in memory retrieval, resulting in enhanced adaptability of agents and more effective interactions with their environment and other agents. We evaluated the quality of the retrieved memories using LLMs in a comprehensive test simulation set and conducted a quantitative analysis of agent behavior consistency across various memory retrieval modes. This novel memory retrieval method allows agents to better simulate human-like responses based on their current state, thereby significantly improving their ability to engage in complex interpersonal interactions.

In summary, our paper makes the following contributions:

- We constructed a novel, text-based generative agent simulation environment, featuring multiple characters and locations, which simulates real-life interactions at a low computational cost, demonstrating a novel application of LLMs in simulating human-like agent behavior.
- We introduced an innovative Auxiliary Cross Attention Network for memory retrieval in AI agents simulating human behavior. By calculating attention weights between the agent's current state and all memories in the memory bank, ACAN ranks and retrieves the most relevant memories, leading to Enhanced Memory Retrieval compared to base methods.
- We introduced a novel methodology for training neural networks with LLM assistance, where LLMs evaluate memory retrieval outputs and help in shaping the loss function. This innovative use of LLMs in training AI agents offers a fresh perspective on their application in the AI agent field.
- We provide a detailed comparison between our method and commonly used memory retrieval techniques, demonstrating our approach's superior ability to dynamically adapt to the agent's evolving memory and environment. This novel memory retrieval method enables agents to more accurately simulate human-like responses based on their current state, significantly enhancing their capacity to engage in complex interpersonal interactions.

## 2 Related work

### 2.1 Large language models and generative agent

Generative artificial intelligence refers to AI systems that generate text, images, videos, or other data types based on prompts. These systems are exemplified by LLMs such as ChatGPT and GPT-4, which have achieved tremendous success across various tasks in the field of Natural Language Processing (NLP) (OpenAI, 2022). The primary feature of LLMs is their use of large-scale datasets to train large-scale models, such as GPT-3 (Brown et al., 2020), a precursor to ChatGPT, which was trained using massive data and the Transformer architecture (Vaswani et al., 2017).

The success of OpenAI's ChatGPT has sparked considerable interest among researchers as a potential spark for Artificial General Intelligence (AGI) (Xi et al., 2023). Numerous studies have validated the exceptional performance of LLMs when appropriately prompted in downstream tasks, showcasing their versatility and intelligence (Bang et al., 2023; Wei et al., 2022). These models have been effectively employed in a variety of applications, such as translation (Jiao et al., 2023), text generation across diverse genres (Cao et al., 2023), and narrative content adaptation (Musacchio et al., 2024).

A particularly notable application is the development of AI agents capable of mimicking human behaviors using ChatGPT, which illustrates the models' capability to generate believably human-like interactions (Xi et al., 2023).

In the broadest sense, an “agent” is defined as an entity capable of action (Zalta et al., 1995). Within the field of artificial intelligence, there has been a longstanding commitment to using agents as believable proxies for human behavior, a goal that holds significant importance across AI and its applications (Bates et al., 1994; Laird and VanLent, 2001; Yannakakis, 2012). Typically, AI-based agents are designed to perceive their environments through sensors, make decisions, and perform actions using effectors (Wooldridge and Jennings, 1995; Russell and Norvig, 2016). These agents combine sensory data with pre-programmed behaviors to interact with their surroundings effectively, but creating agents that truly mimic the nuanced behaviors of humans remains a complex endeavor.

Despite this, the development of AI agents that can accurately and credibly simulate complex human behaviors has proven to be challenging (Schweitzer et al., 2020; Abdalla and Mishra, 2021). Currently, the widespread success of LLMs, exemplified by GPT-4, across various AI domains (Achiam et al., 2023), has enabled these models to leverage extensive training data on human behavior (Brown et al., 2020), providing agents with enhanced creativity and adaptability. This capability enables agents to process information more effectively and respond in ways that closely mimic human reactions. Consequently, an increasing number of researchers are exploring the use of LLMs to develop Generative Agents with robust content generation capabilities (Park et al., 2023; Liu et al., 2023; Wang et al., 2024). These agents are finding applications in diverse fields, demonstrating the versatility and potential of LLM-driven agent applications.

LLM-based agents primarily utilize prompt chains (Wu T. et al., 2022) to generate concise natural language descriptions and actions for characters within prototype systems, thereby creating populated prototypes for social computing systems (Park J. S. et al., 2022). Additionally, LLMs are employed to craft interactive experiences in user-engaging games, facilitating dynamic actions (Freiknecht and Effelsberg, 2020) and text-based adventure games (Callison-Burch et al., 2022). Further extending their application, LLM-driven Generative Agents are used to construct virtual communities. Within these simulated environments, researchers have observed social phenomena emerging from the cooperation among multiple agents (Park et al., 2023). For instance, in a virtual community, an agent planning a Valentine's Day party autonomously spreads invitations throughout the community and coordinates the timing of the event over the following two days.

To enable Generative Agents, assisted by LLMs, to perform such complex functions, researchers have explored methods beyond first-order prompting. They have enhanced language models with static knowledge bases and information retrieval schemes (Khattab et al., 2022), and extended these concepts to develop agent architectures that dynamically update past experiences at each step, integrating these with the agents' current contexts and plans. For instance, applications in various domains utilize such memory-enhanced agents to process layered information and improve decision-making (Yu et al., 2024). This integration can either reinforce or contradict the ongoing interactions, providing a more adaptive and responsive agent behavior (Park et al., 2023).

However, the complex behavior of agents inevitably leads to challenges similar to human decision-making, particularly the need for an appropriate memory system. This system must enable agents to retrieve the most relevant memories when needed, thereby facilitating recollection and thought processes akin to human cognition. Without such a system, agents may exhibit inconsistent behaviors over time, undermining the believability and effectiveness of their interactions.

### 2.2 Agent memory retrieval

In constructing memory systems for Generative Agents, agents' memories—comprised of sequences of past observations, thoughts, and actions (Nuxoll and Laird, 2007)—play a crucial role in strategy formulation and decision-making processes. Just as the human brain utilizes prior experiences for adaptive behavior (Squire, 1986; Schwabe et al., 2014), agents require specialized memory mechanisms to effectively manage sequential tasks. Research by Schuurmans (2023) demonstrated that transformer-based large language models (LLMs), when augmented with external memory, achieve computational universality. This augmentation allows agents to revisit and reapply past strategies without altering the model's weights, which is critical for reliable adaptation in complex environments.

Before the advent of LLM-based agents, extensive research had already been conducted on enhancing model performance through memory mechanisms. For instance, Memory Transformer and Recurrent Memory Transformer (Burtsev et al., 2020; Bulatov et al., 2022) introduced memory tokens and recurrent mechanisms to improve transformers' understanding of long-sequence tasks, especially for global context processing. Memorizing Transformers (Wu Y. et al., 2022) leveraged non-differentiable memory lookup systems to retrieve past inputs during inference, enabling real-time memory retrieval. Additionally, hardware-related research has explored optimizing memory utilization to improve model efficiency (Sridharan et al., 2023). However, these memory mechanisms primarily targeted deep learning models, optimizing performance through memory augmentation or architectural adjustments within a fixed model framework.

In contrast, LLM-derived agents, functioning as independent entities, face a more complex and dynamic memory landscape. These agents do not rely solely on internally generated representations from training, but also draw heavily from their interaction history and external memory repositories. For example, Memory Sandbox (Huang et al., 2023) introduced a system where users can manage conversational memories of LLM-powered agents, treating them as data objects that can be viewed, manipulated, and controlled, thus enhancing interaction transparency and coherence. Similarly, AgentSims (Lin et al., 2023) provided a sandbox infrastructure for task-based evaluations of LLM agents in simulated environments, giving researchers a platform to test memory and planning mechanisms in LLMs. A recent survey (Zhang et al., 2024) further highlights the significance of memory modules in enabling LLM-based agents to achieve self-evolving capabilities and interact effectively in real-world contexts. Furthermore, the Retrieval-Augmented Planning (RAP) framework (Kagaya et al., 2024) leverages contextual memory to enhance decision-making in both text-based and multimodal environments.

Enhancing memory retrieval in generative agents not only improves LLM performance but also enhances the extraction of external memories, thereby boosting the agents' behavior and adaptability. This is particularly critical in multi-agent systems, where each agent may have distinct external memory structures, making efficient retrieval essential. The primary method for memory utilization in LLM-based agents involves using relevant memories as prompts. However, as agents accumulate more historical data through interactions, two major challenges arise. First, the length of these records may exceed the processing limits of the LLM's Transformer architecture, causing content truncation. Second, the growing volume of observations and actions complicates the retrieval of relevant memories, leading to potential misalignment between the agent's responses and the current context. Addressing these challenges requires the development of efficient memory retrieval systems capable of managing and utilizing extensive historical data in a way that maintains coherence and relevance in the agent's interactions.

To address these challenges, current improvements in agent memory management include techniques such as text truncation (Park H. H. et al., 2022), input segmentation (Mohtashami and Jaggi, 2023), and other approaches aimed at reducing complexity, such as increasing the sequence length limits of Transformer-based LLMs (Guo et al., 2021), or incorporating self-controlled memory systems to manage long-term and short-term memory efficiently (Liang et al., 2023). Furthermore, methods for integrating and summarizing memories to create condensed representations have been developed (Zhao et al., 2024; Liang et al., 2023), enhancing the efficiency of memory retrieval in dynamic and complex interaction scenarios. Retrieval models such as Alonso et al. (2024) integrate chained-of-table search, vector-database retrieval, and prompting mechanisms to handle time-sensitive and context-dependent queries. Similarly, Hou et al. (2024) propose a human-like memory architecture for LLM-based dialogue agents, leveraging cue-based recall and a mathematical model for dynamic memory consolidation, enabling temporal and context-sensitive retrieval. Additionally, data structures and embedding techniques have been explored to compress memories, facilitating faster response times in interactions (Modarressi et al., 2023; Qian et al., 2023), while SQL-integrated systems enable efficient management of large-scale historical data through SQL commands (Hu et al., 2023; Zhou et al., 2023).

In multi-agent systems, when agents interact with their environment and other agents, the ability to retrieve the most relevant information from their memory is essential. Particularly in environments that require collaboration among multiple agents, the quality of memory retrieval significantly influences the agents' decision-making, actions, and adaptability. This crucial aspect of memory optimization is aligned with the objectives of multi-agent reinforcement learning (MARL), where enhancing agent capabilities is a primary focus (Gronauer and Diepold, 2022). For example, the introduction of memory-driven communication mechanisms via memory devices has enabled agents to share and update information about their environment during task execution, significantly improving coordination and performance in complex multi-agent systems (Pesce and Montana, 2020).

However, unlike traditional MARL approaches that primarily utilize memory for storing learned policies or state-action histories, LLM-based multi-agent systems rely on pre-trained models, and their intelligence is not updated through real-time training as in MARL. In MARL, agents continuously improve by interacting with their environment, refining their strategies via reinforcement learning. In contrast, LLM agents depend on external, evolving memory banks to access accumulated historical interactions. The focus thus shifts from real-time learning to optimizing memory retrieval, as these external memories are queried in real-time. ACAN enhances LLM agents by improving how relevant memories are retrieved, allowing for more effective decision-making and adaptability in complex environments. MemoryBank (Zhong et al., 2024) exemplifies this, using past interaction data and the forgetting curve theory to optimize memory retrieval. Similarly, advanced methods use metrics like Recency, Relevance, and Importance to dynamically rank and retrieve the most suitable memories (Park et al., 2023), underscoring the importance of adaptive memory systems in evolving agent environments.

In summary, the literature review underscores the critical role of memory in enhancing the capability and adaptability of agents within multi-agent systems. The efficacy of generative agents in practical applications is directly determined by the capability of memory retrieval systems to extract the most relevant memories from the memory bank, akin to human-like recollection based on the current context faced by the agent. However, current methods of memory retrieval still struggle to perfectly extract the most relevant memories from the memory bank as a human would, based on the agent's current scenario.

## 3 Methods

To validate the effectiveness of our proposed Auxiliary Cross Attention Network for agent memory retrieval, we have structured the experimental section into distinct parts. The first part details the operational architecture of our text-based generative agent community, which is powered by ChatGPT. The second part describes the structure and training methodology of the Auxiliary Cross Attention Network. Together, these sections provide a comprehensive overview of the experimental framework.

### 3.1 Generative agent architecture

To construct a virtual agent community for testing memory retrieval mechanisms, we simplified the structure described in Park et al. (2023) and proposed a purely text-based community architecture without visual imagery. This setup allows for the instantiation of maps and unique agent entities, where the locations on the map and the number and characteristics of each agent, including their professions and personalities, can be freely defined. We adopted the same GPT-3.5-turbo version of ChatGPT used in Park et al. (2023) to generate agent behaviors. This approach ensures that the comparison of memory retrieval performance is not influenced by variations in the capabilities of the large language models.

In our community architecture, we designated eight agents, each assigned a representative occupation: Farmer, Grocer, Doctor, Mayor, Chef, Hunter, Blacksmith, and Carpenter. Each agent was also given a personality description relevant to their profession. This configuration enriches the complexity of the agent community while balancing the time required for simulation.

Furthermore, in terms of the map design, we allocated a specific home and workplace for each agent, along with a corresponding functional description to ensure alignment with the agent's profession. For instance, the agent with the occupation “Farmer” has “Fields” as their workplace, while the “Doctor” is associated with the “Clinic” as their workplace. Beyond the individual homes and workplaces of each agent, the map includes several communal locations such as the “Village Square” and “Playground” to enhance interactions among the agents.

**Figure 2:**
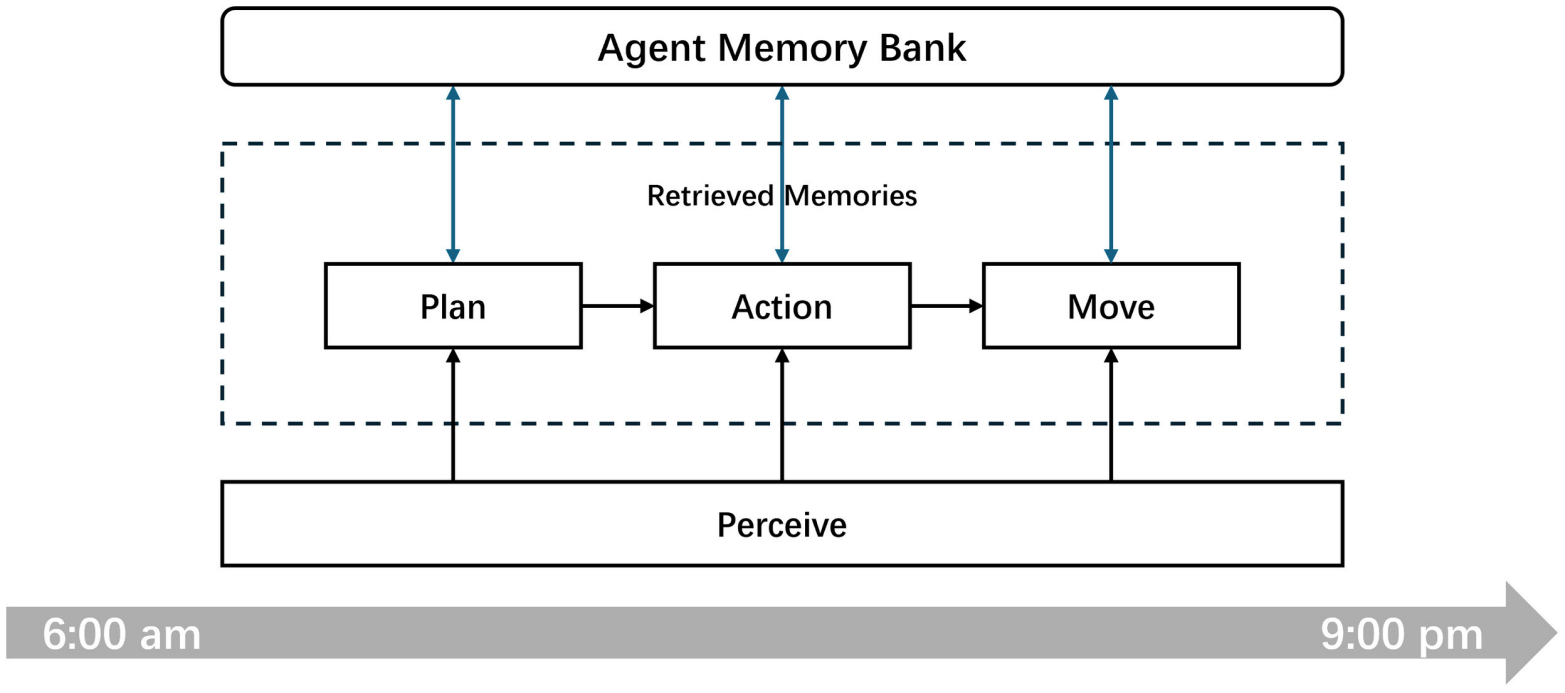
Discrete time cycle of agent activities in the simulated community. The diagram illustrates the daily schedule from 6:00 AM to 9:00 PM, delineating the key activities: planning, action, and movement, underpinned by continuous perception.

As illustrated in Figure 2, to streamline the simulation process, we structured the map and temporal dimensions using a discretized, turn-based format. Temporally, we divided each day within the simulated community into 16 active hours, from 6:00 AM to 9:00 PM, with the remaining hours allocated for sleep. During each hour, agents sequentially act based on their current states and observations. They plan their actions, decide whether to interact with other agents at the same location, and upon completing their actions, they determine their next destination based on the outcomes and their current states. This setup ensures a controlled environment where the impact of agent interactions and decision-making processes can be methodically analyzed.

During the simulation process, memory preservation and retrieval are integral to every action undertaken by an agent. Each time an agent's plan or action is determined through a prompt processed by the LLM, it requires extracting relevant memories from the agent's memory bank using a memory retrieval algorithm. This retrieval is based on the current state query to decide the agent's subsequent actions. Additionally, after each action, agents store their experiences, actions, and observations back into the memory bank, enhancing the resources available for future memory retrieval. Thus, under these simulation conditions, the critical role of the memory retrieval algorithm is further emphasized, highlighting its importance in the functionality and effectiveness of the generative agents.

### 3.2 Auxiliary cross attention network for memory retrieval

In this section, we outline our methodological approach step by step, including the algorithmic details necessary to train the Auxiliary Cross Attention Network for Memory Retrieval. To commence training, we must first generate the requisite training data through simulation. Both the simulation and the subsequent training phases require a foundational memory retrieval system to facilitate the agent's memory recall processes effectively.

Existing memory retrieval methods for agents primarily focus on relevance and temporal validity. For instance, Hou et al. (2024) propose a human-like memory architecture with cue-based recall and dynamic memory consolidation. Retrieval models such as Alonso et al. (2024) employ vector-database mechanisms to handle time-sensitive and context-dependent queries. Similarly, Park et al. (2023) introduce a generative memory scoring framework that balances multiple retrieval criteria. To address these aspects comprehensively, we have implemented a unified memory scoring method, Weighted Memory Retrieval (WMR), as our baseline memory retrieval approach, which calculates memory retrieval scores based on the following criteria:


(1) $$\begin{array}{r} \text{WMR}\left(m\right)=w_{r}\cdot \text{Recency}\left(m\right)+w_{i}\cdot \text{Importance}\left(m\right)\\ +w_{s}\cdot \text{Relevance}\left(m,q\right), \end{array}$$

In this formulation, *Recency* represents the memory decay score, which decreases hourly by a decay factor of 0.995. The *Importance* score is generated by LLM, determining the agent's perceived significance of the memory. *Relevance* measures the cosine similarity between the embedding vectors of each memory in the memory bank and the current state's query embedding. This is mathematically expressed as:


(2) $$\begin{array}{l} \text{Relevance}\left(m,q\right)=\frac{m\cdot q}{\left|m||q\right|} \end{array}$$

where *m* is the memory embedding vector and *q* is the query embedding vector.

After applying this scoring system, the memories with the highest scores are selected, and the top *k* memories are retrieved as the base memory set $m_{r}^{\prime }$.

During the training of our Auxiliary Cross Attention Network, as detailed in Algorithm 1, we systematically employ a dataset consisting of the states *q* faced by each agent during decision-making and the associated memory bank *M* collected during the simulation. The decision-making contexts and the corresponding memory banks of each agent are converted into high-quality text embeddings using the text-embedding-ada-002 model provided by OpenAI (Neelakantan et al., 2022). This model ensures the embeddings preserve the semantic richness essential for effective training. The algorithm iteratively adjusts the network weights to optimize the retrieval of relevant memories based on the agents' current contextual needs. This optimization is facilitated by a cross attention mechanism that aligns the agent's query with the most relevant information from the memory bank.

**Algorithm 1:** Training of the cross attention network for memory retrieval.

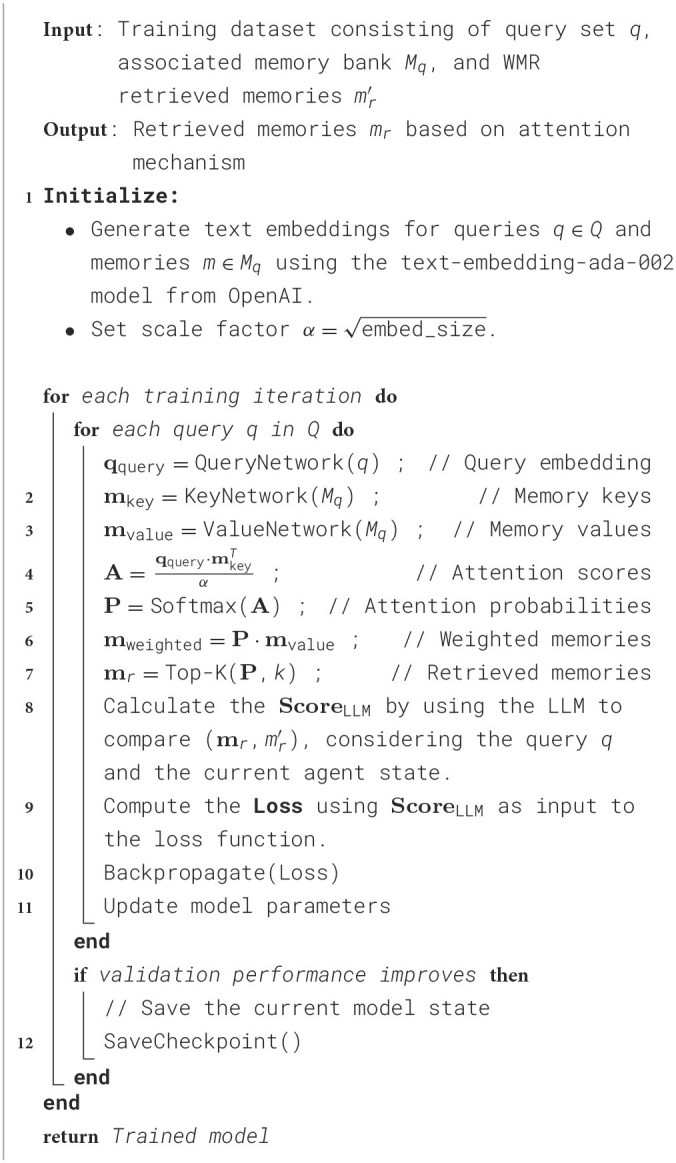

Furthermore, drawing upon research in explainable deep learning (Serrano and Smith, 2019), we innovatively decided to determine the output of the model, specifically the retrieved memories, not by the weighted memories vector *m*_weighted_, but rather through the model's cross attention mechanism. The computation of attention probabilities is defined by the following equation:


(3) $$\begin{array}{l} \text{P}=\text{Softmax}\left(\frac{{\text{q}}_{\text{query}}\cdot {\text{m}}_{\text{key}}^{T}}{\alpha }\right) \end{array}$$

This change emphasizes the importance of interpretability in memory retrieval, allowing for a clearer understanding of how and why certain memories are retrieved based on the agents' current queries.

To further refine our model's memory retrieval capabilities, the cross attention weights across different memories in the memory bank are ranked, and the top-*k* memories are selected as the output *m*_*r*_ for the model's retrieved memory.

To ensure that retrieved memories effectively guide an agent's behavior during training, it is essential to assess their quality. While human evaluation is often considered the gold standard, it can be impractical due to its time-consuming nature, higher costs, and potential variability among evaluators. Recent studies have demonstrated that large language models (LLMs) can serve as reliable evaluators for natural language generation (NLG) tasks, exhibiting strong correlation with human judgments. For instance, Wang et al. (2023) found that ChatGPT achieved state-of-the-art or competitive correlation with human evaluations across various NLG tasks. Similarly, Chiang and Lee (2023) showed that LLM-based evaluations were consistent with expert human assessments in tasks such as open-ended story generation and adversarial attacks. Given the dynamic nature of agent interactions, LLMs offer a consistent and scalable method for evaluating memory relevance, effectively considering context and quality.

Therefore, we employ an LLM to compare and score the memories retrieved by our model, denoted as *m*_*r*_, against those retrieved using a baseline method, denoted as $m_{r}^{\prime }$. This comparison is based on the current state of the agent and the memory query state, denoted by *q*. The LLM evaluates the relevance of *m*_*r*_ and $m_{r}^{\prime }$ to the agent's current state and query *q*, assigning scores based on their contextual appropriateness and alignment with the agent's goals on a scale from 1 to 10, producing scores *Score*_LLM_ and $Score_{\text{LLM}}^{\prime }$, respectively. The following loss function is then computed to train the model effectively:


(4) $$\begin{array}{l} \text{output\_score}=\frac{Score_{\text{LLM}}-Score_{\text{LLM}}^{\prime }}{10} \end{array}$$

(5) $$\begin{array}{l} \text{loss}=\max\left(-\log\left(\text{output\_score}+1\right),0\right) \end{array}$$

The cross attention mechanism within our model dynamically ranks and retrieves memories based on their relevance to the given query *q*, leveraging the current agent state for context. This process not only enhances the responsiveness of the model to the evolving scenario within the agent environment but also aligns the retrieved memories more closely with the needs of the agent.

The loss function of our model is meticulously designed to optimize memory retrieval capabilities. It is defined as the logarithm of the normalized difference between scores assigned to the model-generated and baseline memories, effectively penalizing deviations from expected outcomes. This approach ensures the model not only learns to accurately retrieve relevant memories but also continually refines its retrieval process based on ground truth data, enhancing its adaptability in real-world scenarios.

To support this advanced training approach, the model parameters are finely tuned using the Adam optimizer. This optimizer is chosen for its ability to efficiently manage sparse gradients and adaptively adjust learning rates, which are vital for quickly converging to the most effective solutions.

The integration of a cross attention network, optimized through the use of large language models, further enhances the model's memory retrieval capabilities. This setup improves the efficiency and relevance of how memories are accessed within generative agents, leveraging the computational power of LLMs to refine the training process effectively. The use of LLMs to guide the training process allows our model to operate effectively with the support of advanced AI technologies, thereby making a significant contribution to the field of AI-driven memory management.

## 4 Results

### 4.1 Result analysis of auxiliary cross attention network

For the generation of our training dataset, we simulated the behavior of a pre-defined community of eight agents over three consecutive days, each consisting of 16 h of interactions. During these simulations, agents engaged in various tasks, similar to the agent-based interactions described in Generative Agents (Park et al., 2023). Each agent's behavior was guided by ChatGPT (GPT-3.5-turbo), which generated context-specific interactions and stored the outcomes as memories. At every decision-making step, the agents' current state, past memories, and retrieved memories [ranked by the Weighted Memory Retrieval (WMR) method] were saved in the memory bank and vectorized using the text-embedding-ada-002 model, producing an embedding size of 1,536. Each training data entry included the agent's current state *q*, the corresponding memory bank *M*_*q*_, and the WMR retrieved memories $m_{r}^{\prime }$ ranked based on Recency, Importance, and Relevance.

The structure of a single training entry consisted of the agent's query (current state), the action taken, the type of action (e.g., interaction, decision), the prompt guiding the action, and the retrieved memories at that point in time. This complete data structure captures how an agent's decision is informed by both past experiences and context-specific information, ensuring a comprehensive training process. In total, 1,280 unique training entries were generated, each encapsulating the dynamic interaction between the agents and their environments, enhancing data diversity and robustness.

Once the training dataset was prepared, we configured the training parameters for the memory retrieval model. We used the Adam optimizer with a learning rate of 0.001 and a batch size of 16, while the text embeddings for memory were fixed at a size of 1,536. During retrieval, the model output the top five memories ranked by attention weights. The entire training process was executed on an NVIDIA RTX 4060 GPU, which significantly accelerated the model's convergence. Each agent's interaction data, including the current state, query, and retrieved memories, were incorporated into the model to optimize the memory retrieval process for generative agents in multi-agent settings.

**Figure 3:**
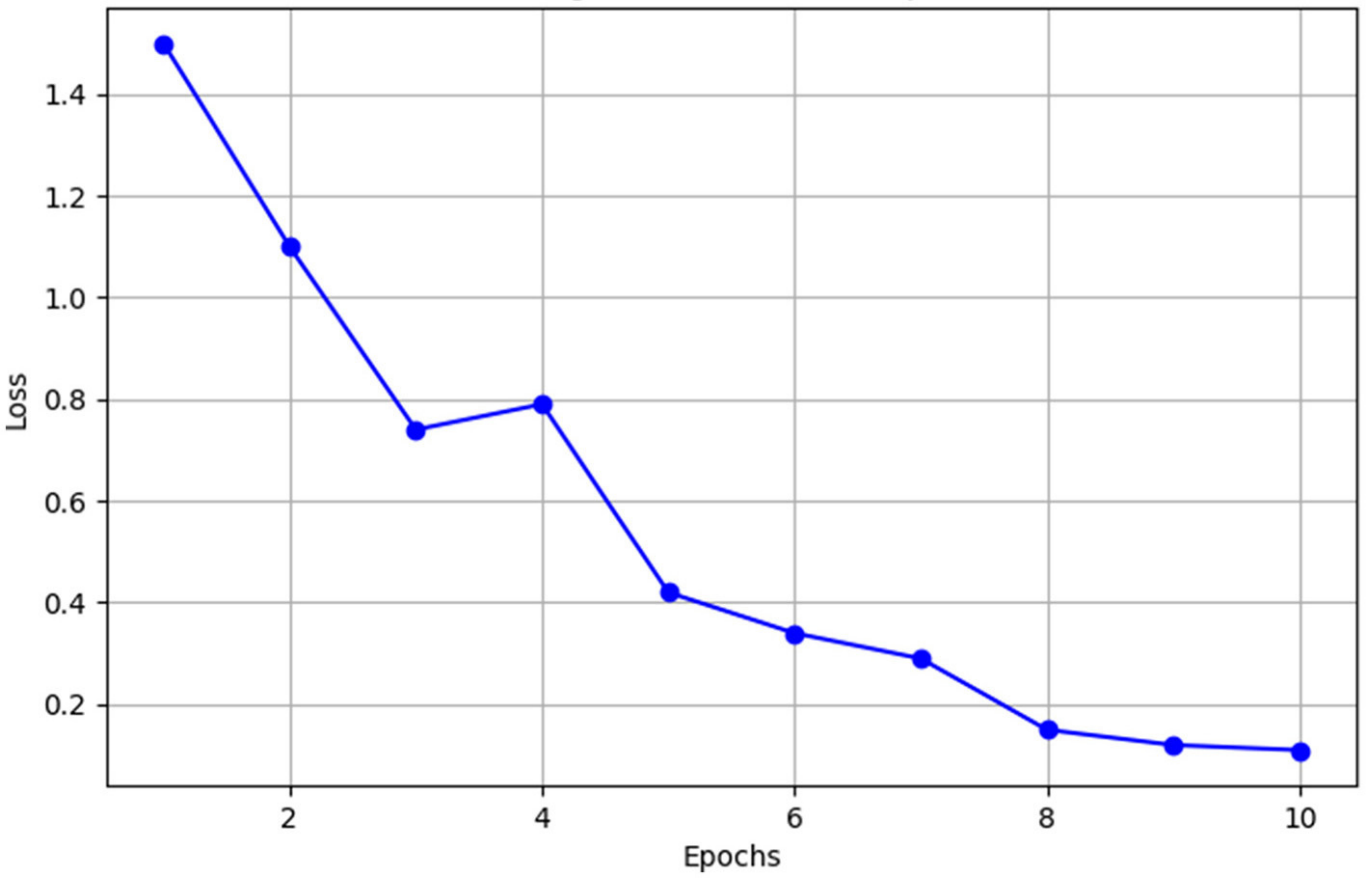
Training loss curve over epochs.

The effectiveness of the model's training under the assistance of a LLM is demonstrated in Figure 3. This figure illustrates the significant decrease in training loss across epochs.

As illustrated in Figure 3, the model demonstrates significant improvement under the guidance of LLMs. The training loss declines sharply from an initial value of 1.5 to 0.12. This reduction is driven by the loss function, which incorporates scores provided by the LLM based on the agent's current context, to assess the memories retrieved by both the proposed and baseline methods. This downward trend indicates the model's increasing effectiveness in adapting to the data, optimizing parameter adjustments to better capture and utilize representative memories. Consequently, this enhancement enables the model to consistently outperform the baseline method in memory scoring, contributing to the significant reduction in loss.

To rigorously evaluate the performance of our proposed model, we conducted test simulations spanning a complete day, covering 16 h, using both the WMR memory retrieval method and the ACAN memory retrieval method based on the fully trained Auxiliary Cross Attention Network. The test involved eight agents, each representing different professions and personalities, consistent with the setup used during the training phase. These simulations generated a total of 435 data entries for comparative analysis. Given the nascent stage of research in this area, particularly regarding LLM-based generative agents, the baseline memory retrieval method we used Park et al. (2023) represents one of the most state-of-the-art approaches currently available for comparison in agent memory retrieval. This ensures a fair and meaningful benchmark against which the performance of our ACAN model could be evaluated.

The assessment of the test data was conducted in the same manner as during training, where a large language model was employed in conjunction with the agent's contextual state to score the memories generated during the simulation on a scale from 1 to 10. We compared the memory retrieval scores from the ACAN model with those retrieved using the WMR memory retrieval method across all test data.

**Figure 4:**
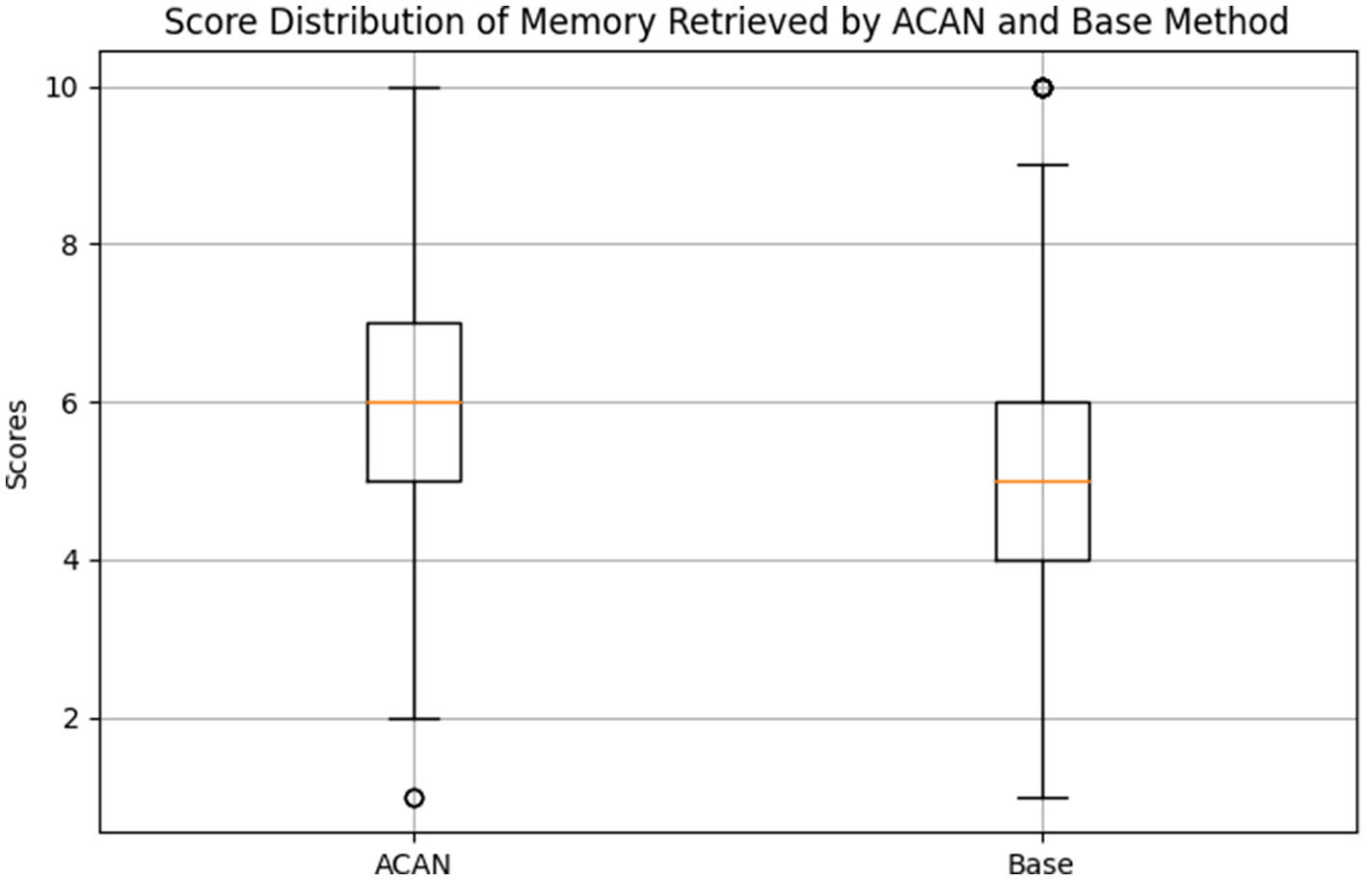
Score distribution of memory retrieved by ACAN and weighted memory retrieval (WMR).

As illustrated in Figure 4, the results of memory retrieval using the ACAN model in comparison with the WMR method show that the ACAN method consistently achieves higher memory scores than the baseline. Specifically, the ACAN group scored an average of 5.94 with a standard deviation of 1.66, whereas the baseline group scored an average of 5.05 with a standard deviation of 1.88. Statistical tests further validate the significance of these differences, with a T-statistic of 7.44 and a corresponding P-value of 2.42 × 10^−13^, significantly below the common significance level of 0.05. This strongly indicates that the ACAN model substantially outperforms the baseline method in terms of memory retrieval effectiveness.

The superior performance of the ACAN model can be attributed to its dynamic cross-attention mechanism, which optimizes memory retrieval by continuously adapting to the agent's evolving state and context. This mechanism allows the ACAN model to rank memories based not only on basic relevance metrics such as recency but also on a more nuanced evaluation of the importance of past experiences, as influenced by real-time feedback from the LLM. In contrast, the WMR method relies on static retrieval strategies that do not account for these contextual factors, leading to less accurate and less relevant memory retrieval.

Furthermore, the reduced standard deviation in the ACAN results indicates that the model consistently performs well across different scenarios, demonstrating its robustness in diverse environments. The WMR method, with a higher standard deviation, shows more variability in its effectiveness, suggesting that its performance is more dependent on specific scenarios or task conditions.

These findings also have broader implications for agent behavior and decision-making. By retrieving more relevant and contextually appropriate memories, the ACAN model enhances the agent's ability to make informed decisions that closely mimic human-like responses. This, in turn, improves the quality of the agent's interactions with both the environment and other agents. The results provide empirical support for the hypothesis that the ACAN model's memory retrieval mechanism leads to more natural and effective decision-making processes in multi-agent settings.

In addition, a deeper analysis of the memory scores reveals that the ACAN model particularly excels in scenarios that require the integration of complex, long-term memories. This suggests that the model's cross-attention mechanism not only improves short-term relevance but also facilitates the retrieval of critical long-term memories that might otherwise be overlooked in traditional retrieval methods. This highlights the potential for the ACAN model to enhance not only immediate decision-making but also more complex tasks involving strategic planning and social interactions.

### 4.2 Quantitative analysis of memory retrieval

While the training of our model and the assessments on the generated test set were conducted with the support of LLMs, we aimed to analyze the effectiveness of our proposed memory retrieval method without direct LLM intervention. For this purpose, a quantitative analysis experiment was designed, where agents received specific invitations under different memory retrieval modes, and their attendance probabilities were compared. This experimental setup allows us to evaluate how different memory retrieval strategies impact the agents' perception of external stimuli and their cognitive ability to mimic human behavior.

For the experimental design, each day, random agents were invited at a specified hour to attend events at designated locations, occurring for 10 h excluding sleep times. Following the agents' agreement to attend, their actual appearance at the event at the appointed time was recorded. This simulation was conducted over a span of ten days, involving eight agents under two different memory modes, generating a total of 100 invitation and attendance records.

As shown in Table 1, we compared the memory retrieval effectiveness of the ACAN model and the WMR method across five trials. In these experiments, agents using the ACAN model adhered to invitations 32.6% of the time on average, whereas agents using the WMR method adhered 24.6% of the time. This indicates that agents employing the ACAN model have a significantly higher likelihood of attending the events, with an average attendance rate that is eight percentage points higher than that of the WMR method. Additionally, observing the memory retrieval process of the agents revealed that those who successfully attended the events could accurately recall the relevant invitation information, further validating the ACAN model's effectiveness in enhancing memory retrieval accuracy.

**Table 1:** Detailed attendance rates across five trials for ACAN and weighted memory retrieval (WMR).

**Metric**	**ACAN**	**WMR**
Trial 1	35%	27%
Trial 2	36%	29%
Trial 3	29%	19%
Trial 4	32%	23%
Trial 5	31%	25%
Mean attendance rate	32.6%	24.6%
Standard deviation	2.881%	3.847%

To further quantify the statistical significance of this difference, a paired samples *t*-test was conducted. The *t*-test results yielded a T-statistic of 11.31 and a *P*-value of 0.00035, indicating that the observed difference in attendance rates between the two methods is highly significant (well below the common significance threshold of 0.05). This provides strong evidence that the ACAN model substantially improves the agents' responsiveness to invitations and their likelihood of attending events compared to the baseline WMR method.

Additionally, the standard deviations across the five trials show some variability in the results (2.881% for the ACAN method and 3.847% for the baseline method), but the ACAN model consistently outperformed the baseline, demonstrating both its stability and reliability. These findings emphasize the robustness of the ACAN-based memory retrieval approach in enhancing agents' event attendance behavior and improving their ability to respond to interactions within dynamic and complex simulated environments.

These findings demonstrate the robustness of the ACAN-based memory retrieval approach in enhancing agents' event attendance behavior and their responsiveness to interactions within dynamic and complex simulated environments. The integration of cross-attention mechanisms in ACAN likely facilitates better contextual understanding and memory utilization, which in turn leads to more effective decision-making and engagement in scheduled events. Consequently, the higher attendance rates associated with the ACAN model not only reinforce its effectiveness but also highlight its potential to simulate complex human-like social behaviors. This makes ACAN a valuable tool for applications that require nuanced, contextually-aware decision-making, enhancing the capability of agents to navigate and adapt within multifaceted interactive settings.

## 5 Discussion

This study successfully developed and implemented a text-based generative agent simulation world, creating a community with multiple locations and agents that engage in various interactions. Based on this foundation, we designed an innovative memory retrieval system using the Auxiliary Cross Attention Network. This system simulates human behavior by ranking the attention weights between the agent's current state and memories in the memory bank, retrieving the memories most relevant to the current state. To train this model, we introduced an innovative approach by leveraging the assistance of LLMs. During training, the LLM scores the memories retrieved by our model against those retrieved by the baseline method, using these scores along with a novel loss function to train the model effectively.

Our evaluations leveraged a test data set generated from simulations of LLM-based agent interactions, representing a typical day in the life of these agents. This simulated environment, along with our specially designed agent invitation and attendance experiments, provided a robust framework for validating the advantages of our memory retrieval method over traditional approaches. The results from these evaluations confirm that our system significantly enhances the memory retrieval process, thereby supporting more effective decision-making in generative agents. By optimizing how memories are retrieved and utilized, our method allows agents to respond in ways that are more closely aligned with human behavior based on their current state, thereby enriching their ability to engage in and navigate complex interpersonal interactions.

Despite the achievements of our study, there are notable limitations to consider. The model's effectiveness relies heavily on continuous evaluations by Large Language Models (LLMs), increasing computational demands and operational costs due to LLM API token usage. Additionally, LLM feedback slows training, potentially limiting rapid development and scalability. Our evaluation method, dependent on LLMs, may not generalize across different configurations or domains, and using LLMs instead of human assessment for training and testing could affect result rigor and objectivity, raising concerns about robustness and generalizability. However, recent work such as Edge et al. (2024) demonstrates that LLMs can reliably evaluate relevance and faithfulness in RAG systems, supporting their use as cost-effective alternatives to human assessments. To further enhance robustness, especially in nuanced scenarios, incorporating human validation may serve as a valuable complement.

The broader impacts of our Auxiliary Cross Attention Network (ACAN) model extend significantly across the AI discipline, introducing a novel adaptive framework for memory retrieval that not only enriches theoretical models of AI agent interactions but also demonstrates substantial practical applications. Leveraging LLM assessments to shape the loss function during training is an innovative approach that significantly refines the precision of memory retrieval. This advancement holds great promise for revolutionizing human-agent interactions by enabling more natural and complex interpersonal simulations. Future research should focus on further enhancing the model's capabilities through more sophisticated neural network architectures which could advance the state of memory retrieval in AI agents. Additionally, developing autonomous feedback mechanisms will be crucial for advancing AI agents that can adapt independently to dynamic environments, thus pushing the boundaries of what is possible in Artificial General Intelligence. This focus on improving memory retrieval systems directly supports the evolution of more intelligent and responsive AI agents, paving the way for broader and more effective implementations in various AI-based domains.

## 6 Conclusions

This study has introduced the Auxiliary Cross Attention Network (ACAN), a pioneering memory retrieval system for generative agents, showcasing a significant advancement in AI agent driven by large language models (LLMs). ACAN effectively enhances agent adaptability and behavioral consistency by dynamically ranking and retrieving memories based on the agent's current state, thus addressing the critical need for sophisticated memory management mechanisms in Artificial General Intelligence. While the reliance on LLMs for training and evaluating the system poses challenges for scalability and efficiency, it simultaneously highlights the need for innovations that could decrease such dependencies and enhance the autonomy of the system. This research not only demonstrates the potential of ACAN in improving memory retrieval within varied agent interactions but also highlights the broader applicability of LLMs in advancing AI technologies. Moving forward, the focus will be on refining these methodologies to further enhance the capabilities and independence of AI agents in complex environments.

## Data Availability

The datasets presented in this study can be found in online repositories. The names of the repository/repositories and accession number(s) can be found at: https://github.com/HongChuanYang/Training-by-LLM-Enhanced-Memory-Retrieval-for-Generative-Agents-via-ACAN.
